# Breaking of Wavelength-Dependence in Holographic Wavefront Sensors Using Spatial-Spectral Filtering

**DOI:** 10.3390/s23042038

**Published:** 2023-02-10

**Authors:** Nikita Stsepuro, Michael Kovalev, Evgenii Zlokazov, Sergey Kudryashov

**Affiliations:** 1 Lebedev Physical Institute, Russian Academy of Sciences, Leninskiy Prospekt 53, Moscow 119991, Russia; 2 Department of Laser Physics, National Research Nuclear University MEPhI, 31 Kashirskoe Shosse, Moscow 115409, Russia

**Keywords:** phase modulation, spatial light modulator, white light, wavefront sensor, computer-generated hologram

## Abstract

Nowadays, wavefront sensors are widely used to control the shape of the wavefront and detect aberrations of the complex field amplitude in various fields of physics. However, almost all of the existing wavefront sensors work only with quasi-monochromatic radiation. Some of the methods and approaches applied to work with polychromatic radiation impose certain restrictions. However, the contemporary methods of computer and digital holography allow implementing a holographic wavefront sensor that operates with polychromatic radiation. This paper presents a study related to the analysis and evaluation of the error in the operation of holographic wavefront sensors with such radiation.

## 1. Introduction

Currently, wavefront sensors (WFS) are widely used to solve a multitude problems in various fields of physics, namely to control the shape of a wavefront or detect aberrations of the complex field amplitude [1]. These devices include the well-known Shack–Hartmann wavefront sensor [2,3], curvature sensors [4], pyramidal sensors [5,6], and holographic WFSs [7,8,9]. The principle of the first three sensors is based on analyzing the intensity distribution of an image or set of images using a 2D image sensor. In turn, holographic WFSs are built on the principle of converting the phase of a light wave into its intensity due to the spatial filtering of radiation using holograms. Traditionally, holograms were obtained physically by transferring the intensity distribution of the interference pattern to a photographic emulsion [10]. A disadvantage of holographic WFSs based on such holograms is that they cannot detect several complex field amplitude aberrations at once [11].

Therefore, the next stage in the development of holographic WFSs was the use of multiplexed holograms [12,13]. Here, multiplexed refers to a hologram resulting from the recording of two or more holograms in the same area of a photosensitive medium. The use of such elements increases the performance of the method and reduces the sensitivity of the sensor to amplitude and phase fluctuations [14]. However, as it is known from [15], the diffraction efficiency of such holograms decreases in inverse proportion to the square of the number of holograms due to the limitations of the recording media. This leads to a significant reduction in the intensity of the reconstructed waves for each hologram component. In addition, intermodal (crosstalk) noise is generated during the reconstruction of multiplexed holograms, resulting in a reduction of the signal-to-noise ratio in the analysis plane [16].

The next step in improving holographic methods was the use of computer technology to synthesize holograms [17]. With the development of computer and digital holography methods, the process of creating holograms became more digitalized, which led to the creation of new elements, the synthesis of which was fully realized in a computer. Such holograms are called computer-generated holograms (CGHs) [18,19]. Over the past 20 years, spatial light modulators (SLMs) have been increasingly used in optics [20]. They allow discrete modulation of the amplitude or phase component of a light wave with high spatial resolution. The appearance of such devices allowed holographic WFSs to make a significant leap forward, while opening up new opportunities in various fields of science and technology [21].

Almost all of the existing WFS can work only with monochromatic radiation. The methods and approaches used when working with polychromatic radiation impose certain restrictions [22]. Recently however, a fundamental demonstration of a holographic WFS was carried out that could work with polychromatic radiation due to the achromatic properties of the Fourier CGHs [23]. The operation of this holographic WFS was based on the principles of correlation analysis [24]. However, the assessment of the factors affecting the operation of the sensor was not carried out. This paper presents a detailed theoretical and experimental analysis of the features of the operation of such a correlation holographic WFS with polychromatic radiation.

## 2. Materials and Methods

**Figure 1:**
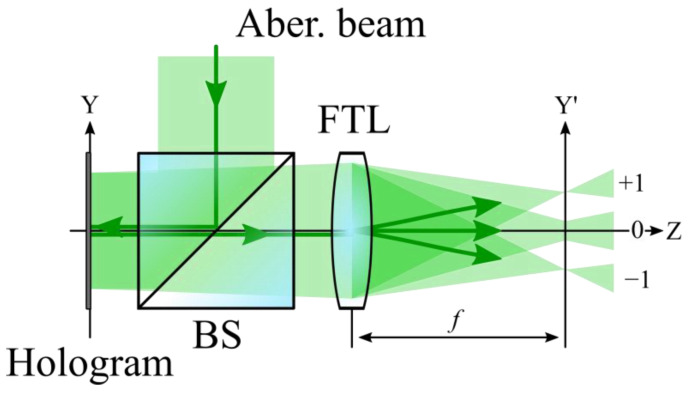
Principle of CGH operation with multiple orders of diffraction.

The principle of operation of the holographic WFS used in the work was as follows: a coherent beam with phase distortion is incident on the Fourier CGH, then the reflection from the SLM radiation enters the aperture of the Fourier Transforming Lens (FTL). This radiation is then focused by the FTL into the plane where the 2D image sensor is installed. In general, when using a Fourier CGH, several diffraction orders are observed in the analysis plane at once [24] (Figure 1). By the intensity and size of these diffraction orders, it is possible to judge the presence and magnitude of the aberration contained in the incident beam of the hologram. When the amplitude of the distortion in the incident light beam coincides with the value of the weight coefficient of the Zernike polynomial recorded in the CGH, an autocorrelation response will be observed in the posterior focal plane of the FTL [25].

The presence of higher diffraction orders in the analysis plane leads to a redistribution of energy between the orders and may cause a decrease in the sensitivity of the method. The solution to this problem was found in the field of computer holography methods, which generally allow increasing the diffraction efficiency due to the formation of only one main diffraction maximum in the Fraunhofer diffraction [26].

This kind of redistribution of energy between diffraction orders in the Fraunhofer diffraction pattern can be achieved using a phase hologram [23,27], whose phase argument can be described by a similar equation

(1) $$h_{ph}\left(x^{\prime },y^{\prime }\right)=\exp\left(j\cdot 2\pi \cdot \left[f_{ref}\left(x^{\prime },y^{\prime }\right)-\frac{\Delta }{\lambda f}y^{\prime }\right]\right).$$

where $f_{ref}\left(x^{\prime },y^{\prime }\right)$ is a spatial phase shift produced by aberration, which is independent from *λ*. It can be presented as:

(2) $$f_{ref}\left(x^{\prime },y^{\prime }\right)=\sum_{n}C_{n}\cdot W_{n}\left(x^{\prime },y^{\prime }\right)$$

where $C_{n}$ is the set of Zernike coefficients, and $W_{n}\left(x^{\prime },y^{\prime }\right)$ is the Zernike polynomials that have been first defined in polar coordinates and then converted into Cartesian coordinates using well-known techniques [28].

During the operation of a holographic WFS, which includes a phase SLM, theoretically the modulation response should be linear and be in the desired range for a specific reference wavelength $\lambda_{0}$ of radiation [29]. To date, there are many methods and approaches for self-calibration of modulators and correction of their gamma curve [30]. After calibration of the SLM for the wavelength $\lambda_{0}$, the phase shift *φ* formed due to the double pass is equal to

(3) $$\varphi \left(\lambda_{0}\right)=\frac{2\pi }{\lambda_{0}}\cdot \Delta n\left(\lambda_{0}\right)\cdot 2d,$$

where $\Delta n\left(\lambda_{0}\right)$ is the refractive index range, and d is the liquid crystal thickness. In the case where the radiation wavelength of the aberration beam does not correspond to the reference wavelength $\lambda_{0}$, the type of dependence of the phase overrun on the SLM parameters will not change

(4) $$\varphi \left(\lambda \right)=\frac{2\pi }{\lambda }\cdot \Delta n\left(\lambda \right)\cdot 2d.$$

If we express d from Equation (3) and substitute it into Equation (4), then the values of phase incursions can be related to each other by the following relation

(5) $$\varphi \left(\lambda \right)=\frac{\lambda_{0}}{\lambda }\cdot \frac{\Delta n\left(\lambda \right)}{\Delta n\left(\lambda_{0}\right)}\cdot \varphi \left(\lambda_{0}\right).$$

**Figure 2:**
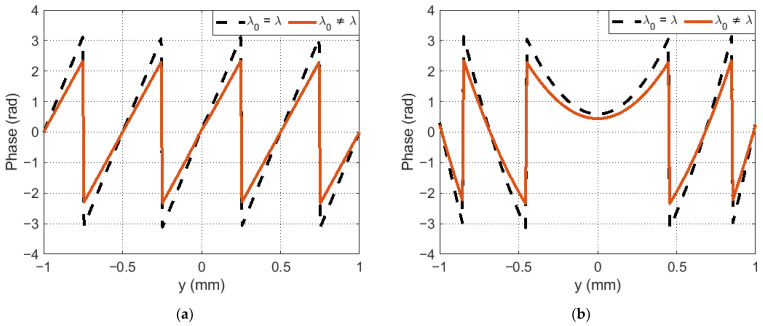
Phase function cross section: (**a**) object wave; (**b**) reference wave in the hologram recording plane.

The discrepancy between the reference wavelength $\lambda_{0}$ and λ leads to a change in the radiation modulation depth. Since the phase function of the reference wave in the Fourier CGH corresponds to the phase function of the one-dimensional blaze grating (Figure 2a), its effective operation requires complete phase modulation of the radiation in the range [0, 2π). If this condition is met, then in accordance with Equation (1), all radiation can be concentrated in the main diffraction maximum [26]. If the condition is not met, this will lead to the appearance of higher diffraction orders. This effect is clearly demonstrated by the example of several distribution periods of the phase argument of the function of the object and reference waves.

Figure 2a shows the phase function profile of the object wave for the case when the reference wavelength is $\lambda_{0}=\lambda$ and $\lambda_{0}\neq \lambda$. By analogy, Figure 2b considers the case for the reference wave in which there is distortion in the form of a defocusing aberration with a weighting factor of 4λ.

It is interesting that Equation (5) does not depend on the chromatic dispersion of the liquid crystal Δn(λ)Δn(λ0) but depends only on the ratio of the wavelengths $\lambda_{0}$ and λ. If we take into account the fact that the effective refractive index range Δn(λ) can be adjusted to the Cauchy dispersion model, then for a certain wavelength λ the relation will have the following form

(6) $$\frac{\Delta n\left(\lambda \right)}{\Delta n\left(\lambda_{0}\right)}≅0.6985+\frac{8.125\cdot 10^{4}}{\lambda^{2}}+\frac{8.125\cdot 10^{5}}{\lambda^{4}}.$$

In this case, the determining factor is that the carrier frequency of the phase jump repetition (i.e., the transition from the maximum to the minimum value) during Fourier CGH modulation remains unchanged even when the radiation wavelength parameter λ is changed. Hence, the modulation depth of the SLM and the Fourier CGH do not affect the principles of correlation response formation. This allows us to extend the functionality of holographic WFSs by replacing the grayscale Fourier CGHs with binary Fourier CGHs and switching from SLM-type devices to digital micromirror devices (DMD)-type, which have much higher operation frequency. It will also greatly simplify the procedure of CGH synthesis used in holographic WFSs, as well as their modulation, as the requirements for phase distribution transfer are significantly relaxed.

## 3. Experimental Demonstration

**Figure 3:**
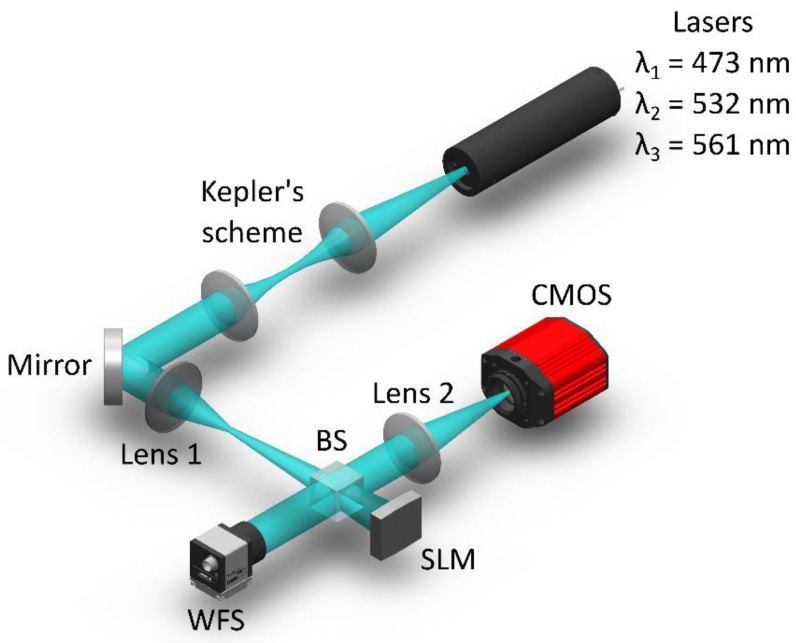
Simplified experimental scheme.

An experimental demonstration of the Fourier CGH properties was carried out using several sources of coherent radiation (lasers with a wavelength of $\lambda_{1}=473$ nm, $\lambda_{2}=532$ nm and $\lambda_{3}=561$ nm), an SLM (Holoeye PLUTO-2-VIS-016, 1920 × 1080, pixel size 8 µm) and a monochrome sCMOS camera (Thorlabs CS2100M-USB, 1920 × 1080, pixel size 5.04 µm). Each of the laser beams was expanded to 4 mm and collimated using a Kepler telescopic system. The convergence of these beams on the same optical axis was carried out using beam-splitting cubes. Figure 3 shows the equivalent optical scheme, which depicts only the main elements of the holographic WFS.

Aberrations of the complex field amplitude were introduced into the optical system using a biconvex lens (Lens 1) with a focal length f=100 mm. Due to the dispersion properties of the lens, the rear focal plane has an extended size along the radiation propagation axis when it is illuminated by radiation with different wavelengths. A Shack–Hartmann wavefront sensor (WFS, Thorlabs WFS300-14AR) with a passport error of λ/50, which measured the value of the aberration of the complex field amplitude in real time (at a frequency of 15 Hz), was used as a control and measuring device. The measurement of aberration by the proposed method was carried out using the SLM, an achromatic FTL (Lens 2) in the range from 0.3 to 0.8 μm and an sCMOS camera, which was located in the rear focal plane of this lens.

**Figure 4:**
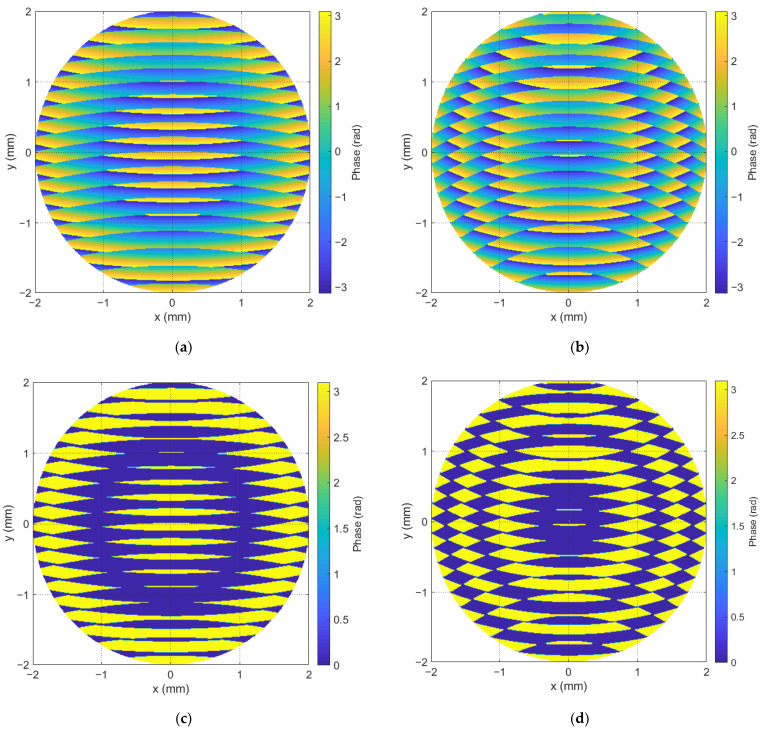
Distribution of the phase argument of the CGH with the value of the weight coefficient in the reference beam: (**a**) 0.53λ for grayscale Fourier CGHs; (**b**) 1.33λ for grayscale Fourier CGHs; (**c**) 0.53λ for binary Fourier CGHs; (**d**) 1.33λ for binary Fourier CGHs.

Holograms were synthesized before the start of the experiment. Then, they were iteratively displayed on the SLM. Each of the holograms was synthesized for one value of the weight coefficient of the Zernike polynomial. Examples of the grayscale Fourier CGHs used in the experiment are shown in Figure 4, where the hologram on the left was synthesized with the value of the weight coefficient in the reference beam equal to 0.53λ, and on the right the value was equal to 1.33λ.

The experiment consisted of two parts, in each of which either grayscale Fourier CGHs or binary ones were used. In each block, the experiment was repeated for each laser separately. In this case, the SLM settings associated with the gamma curve were chosen for the following reference wavelength $\lambda_{0}$: 450 nm, 515 nm, 532 nm and 633 nm. The modulation depth was [0, 2π) at the wavelength $\lambda_{0}$. After the completion of the experimental cycle with grayscale Fourier CGHs, the entire experiment described above was carried out using binary Fourier CGHs. To do this, the previously calculated holograms were binarized using the Otsu method. Figure 4c,d show the binary Fourier CGHs from Figure 4a,b.

**Figure 5:**
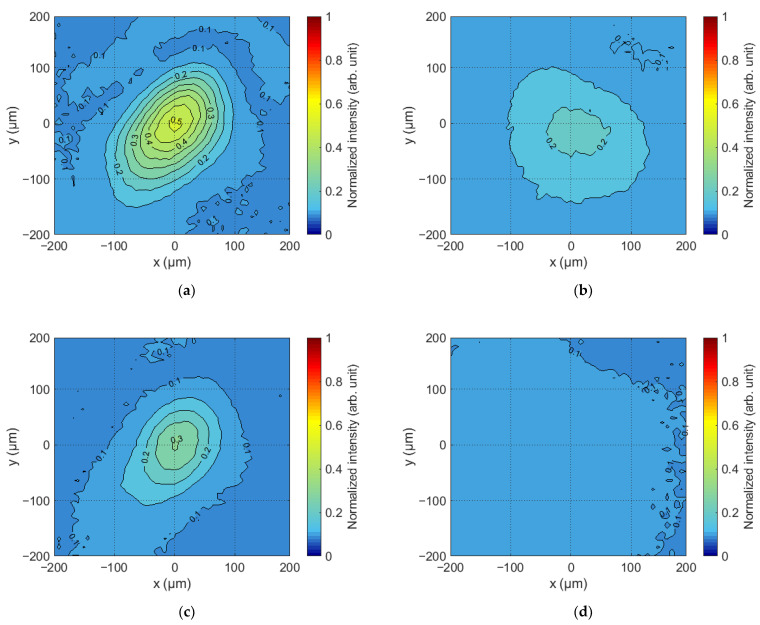
Correlation response in the output to the SLM of the grayscale and binary Fourier CGH with the value of the weight coefficient of the Zernike polynomial: (**a**) 0.53λ for grayscale Fourier CGHs; (**b**) 1.33λ for grayscale Fourier CGHs; (**c**) 0.53λ for binary Fourier CGHs; (**d**) 1.33λ for binary Fourier CGHs.

The output of the holograms on the SLM produced a main diffraction maximum in the rear focal plane of Lens 2 that corresponds to the correlation response. Since the SLM and the sCMOS camera were connected in feedback mode via a PC, it is possible to establish a one-to-one relationship between the maximum intensity of the correlation response and the value of the weight coefficient of the Zernike polynomial recorded in the grayscale Fourier CGHs. Examples of the correlation responses recorded in the experiment are shown in Figure 5. These responses were obtained for the holograms shown in Figure 4. All obtained values were normalized to the maximum intensity value for the selected CMOS camera (I_max_ = 2^16^).

**Figure 6:**
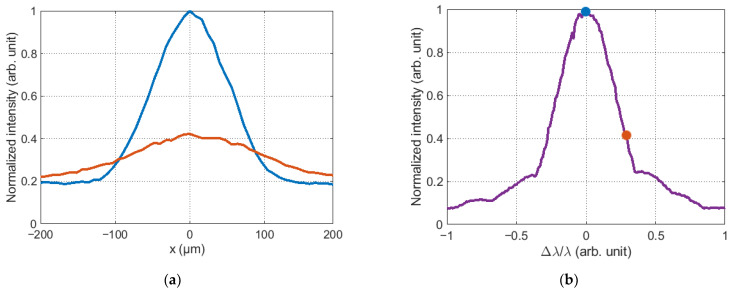
(**a**) Cross section of the correlation function from the Figure 5a,b; (**b**) normalized dependences of the amplitude of the maximum correlation response.

The amplitude of the correlation response varies with the grayscale Fourier CGH output to the SLM. This can be seen in Figure 5. After the intensity distributions were recorded, a global maximum of the two-dimensional distribution was searched using specialized software in the Python environment. The dependence corresponding to the normalized amplitude of the correlation function maxima on the value of the Zernike polynomial weight coefficient was formed on the basis of the obtained values. The maximum value of the function was formed when the value of the weight coefficient of the Zernike polynomial in the CGH Fourier coincided with the value of the aberration of the complex field amplitude in the beam. An example of such a dependence for the defocusing aberration is shown in Figure 6b, where the corresponding colored dots indicate the values of the global maximum taken from Figure 6a.

## 4. Results and Discussion

**Figure 7:**
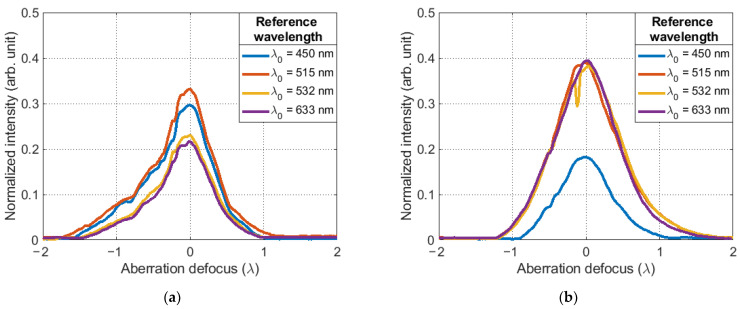

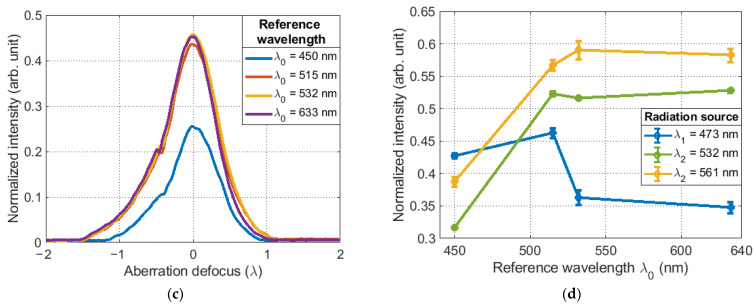
The normalized amplitude of the maximum of the correlation function depending on various parameters of the SLM for sources with a wavelength: (**a**) $\lambda_{1}=473$ nm; (**b**) $\lambda_{2}=532$ nm; (**c**) $\lambda_{3}=561$ nm; (**d**) dependence of maximum correlation function on SLM settings related to reference wavelength $\lambda_{0}$.

The results of the experiments were normalized dependencies of the amplitude of the maximum of the correlation function, the appearance of which is shown in Figure 7. All obtained values were normalized to the maximum intensity value for the selected CMOS camera (I_max_ = 2^16^) and averaged over 10 points.

If we consider the results in detail, we can see that the experimental dependences have a symmetrical profile, one global maximum, a high growth rate of the function and a large signal-to-noise ratio. Their distinguishing feature is only the different value of the maximum of the correlation function. Figure 7a–c show the dependence of the maximum amplitude of the correlation function on the parameters of the SLM, which are associated with the reference wavelength $\lambda_{0}$.

If we assume that the spectral sensitivity of the SLM and the sCMOS camera in the visible range is the same, then this effect can only be explained by the different diffraction efficiency of the Fourier CGHs displayed on the SLM due to phase overmodulation. This leads to the appearance of high-level diffraction orders. Consequently, when they appear, the intensity of the main diffraction maximum will decrease. As a result, the maximum amplitude of the correlation function will change, which is observed in Figure 7d. In the case of using binary Fourier CGHs, the phase function profile of the reference wave changes from oblique to rectangular. Theoretically, in this case only 25% of the energy from the entire light flux can be concentrated in the main diffraction maximum [26].

**Figure 8:**
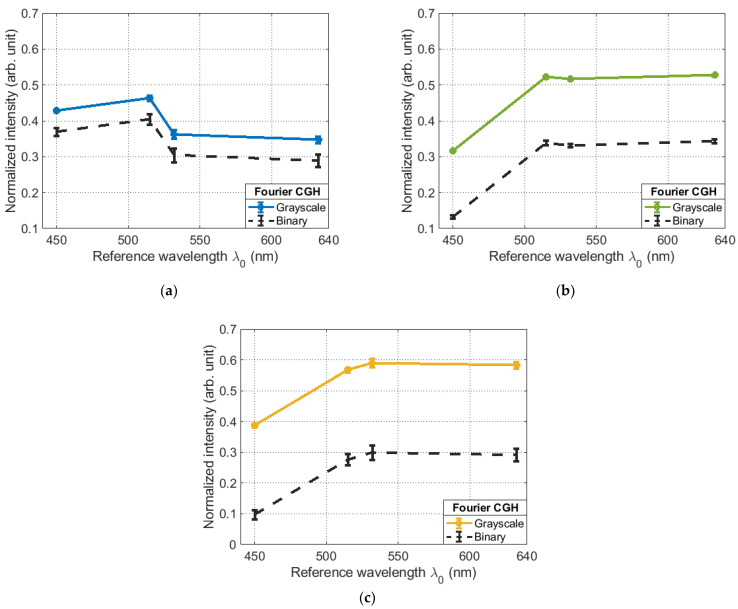
Comparison of the dependence of the maximum of the correlation function on the settings of the SLM relative to the reference wavelength λ_0_ for the grayscale Fourier CGHs and binary at the wavelength: (**a**) $\lambda_{1}=473$ nm; (**b**) $\lambda_{2}=532$ nm; (**c**) $\lambda_{3}=561$ nm.

If you look at the experimental data presented in Figure 8, you can see that when going from the grayscale Fourier CGHs to the binary one, the intensity drop in the main diffraction maximum is not the same for different wavelengths. When calculating the absolute value of the intensity drop for lasers with different wavelengths, the following values were obtained: for $\lambda_{1}=473$ nm, the value was 5.78%, for $\lambda_{2}=532$ nm, the value was 18.44%, and for $\lambda_{3}=561$ nm, the value was 29.08%.

**Figure 9:**
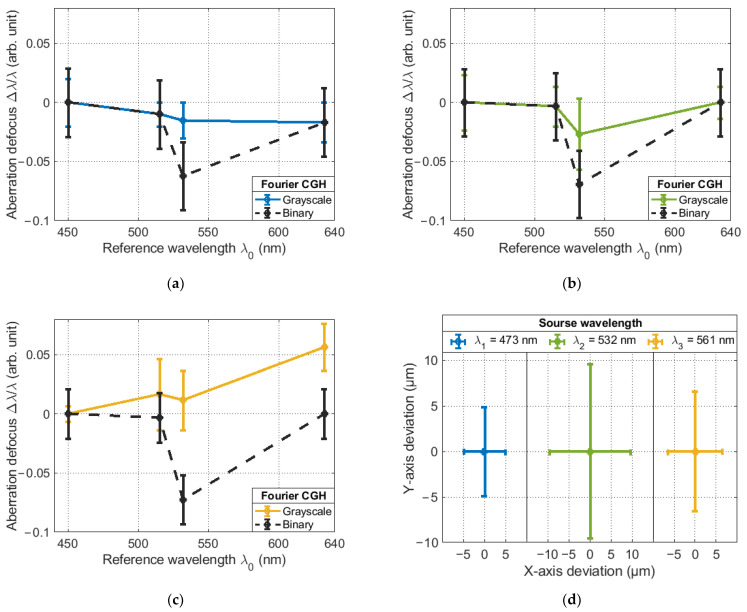
Deviation of the value of the weight coefficient of the Zernike polynomial as a function of the SLM tuning associated with the reference wavelength $\lambda_{0}$ for sources with a wavelength: (**a**) $\lambda_{1}=473$ nm; (**b**) $\lambda_{2}=532$ nm; (**c**) nm; (**d**) the deviation of the position of the correlation response in the analysis plane.

Changing the value of the main maximum of the correlation function does not affect the accuracy of the method. This can be verified by looking at Figure 9, which shows the results of the method’s accuracy assessment. The results have been normalized to the first experimental value for each case to simplify the visualization process.

From the results obtained, it follows that the best result was obtained for the laser with wavelength $\lambda_{1}=473$ nm (Figure 9a), as the maximum deviation of the weighting factor was only λ/31, while for the laser with wavelength $\lambda_{2}=532$ nm and $\lambda_{3}=561$ nm (Figure 9b,c, respectively), the maximum deviation was λ/18 and λ/13, respectively. Meanwhile, the maximum mean deviation when averaged over 10 points was λ/62.5, λ/38.5 and λ/18, respectively. Despite the fluctuations in the weight coefficients of the Zernike polynomials from their true value, their spatial position remains unchanged when the SLM settings are changed with respect to the reference wavelength $\lambda_{0}$. This is evidenced by the results shown in Figure 9d. Assuming a sCMOS camera pixel size of only 5.04 μm, the maximum offsets were 4.88, 9.62 and 6.590 μm. This indicates that the angle of diffraction of the radiation does not change when changing the SLM settings.

## 5. Conclusions

This article shows the influence of the modulation parameters of the used SLM and the obtained grayscale and binary Fourier CGHs when solving the problem of detecting aberrations of the complex field amplitude by the correlation method. The experimental results confirm the theoretical assumptions and prove the applicability of the method when operating in a wide spectral range of wavelengths due to the invariance of the Fourier CGHs themselves to a change in the radiation wavelength of the analyzed wavefront. An analysis of the experimental results shows that with a limited range of phase modulation the method does not lose detection accuracy.

The determining contribution in the proposed method is the profile of the reference wave phase function, which is used in the Fourier CGHs synthesis. The reference wave profile determines the amount of energy in the main diffraction maximum (correlation response) or the value of the signal-to-noise ratio. However, even a significant change in this profile from oblique to rectangular did not distort the results obtained. The replacement of the grayscale Fourier CGHs by binary CGHs allows the DMD to be considered as a phase modulation device, with the advantage of high operating frequencies up to 20 kHz.

Limitations on the spectral range of the studied wave in this method are imposed due to the SLM and the camera. Theoretically, this method is applicable to any spectral range, including UV and IR.

## Data Availability

Data underlying the results presented in this paper are available from the corresponding author upon reasonable request.
